# A Dosimetric Comparison of Dose Escalation with Simultaneous Integrated Boost for Locally Advanced Non-Small-Cell Lung Cancer

**DOI:** 10.1155/2017/9736362

**Published:** 2017-05-28

**Authors:** Wenjuan Yang, Biao Zeng, Yanfang Qiu, Jianfeng Tan, Shilei Xu, Yilong Cai, Yujuan Zhou, Zhigang Liu, Junming Luo, Hui Wang

**Affiliations:** ^1^Key Laboratory of Translational Radiation Oncology, Department of Radiation Oncology, The Affiliated Cancer Hospital of Xiangya School of Medicine, Central South University, Hunan Cancer Hospital, Hunan Province, China; ^2^Department of Radiotherapy Technology, Hunan Cancer Hospital, The Affiliated Cancer Hospital of Xiangya School of Medicine, Central South University, Hunan Province, China; ^3^Department of Pathology, Qinghai People's Provincial Hospital, Xining, Qinghai Province 810007, China

## Abstract

**Background:**

Many studies have demonstrated that a higher radiotherapy dose is associated with improved outcomes in non-small-cell lung cancer (NSCLC). We performed a dosimetric planning study to assess the dosimetric feasibility of intensity-modulated radiation therapy (IMRT) with a simultaneous integrated boost (SIB) in locally advanced NSCLC.

**Methods:**

We enrolled twenty patients. Five different dose plans were generated for each patient. All plans were prescribed a dose of 60 Gy to the planning tumor volume (PTV). In the three SIB groups, the prescribed dose was 69 Gy, 75 Gy, and 81 Gy in 30 fractions to the internal gross tumor volume (iGTV).

**Results:**

The SIB-IMRT plans were associated with a significant increase in the iGTV dose (*P* < 0.05), without increased normal tissue exposure or prolonged overall treatment time. Significant differences were not observed in the dose to the normal lung in terms of the V5 and V20 among the four IMRT plans. The maximum dose (Dmax) in the esophagus moderately increased along with the prescribed dose (*P* < 0.05).

**Conclusions:**

Our results indicated that escalating the dose by SIB-IMRT is dosimetrically feasible; however, systematic evaluations via clinical trials are still warranted. We have designed a further clinical study (which is registered with ClinicalTrials.gov, number NCT02841228).

## 1. Introduction

The prognosis of patients with locally advanced non-small-cell lung cancer (NSCLC) who are not candidates for surgery remains poor. Concurrent chemoradiotherapy has become the first-line treatment [1], and radiation therapy to a total dose of 60 to 70 Gy within 6 to 7 weeks is the standard of care; however, durable local control (LC) is difficult to achieve. Thus, improving LC remains a key issue for patients with stage III NSCLC [2].

An analysis of the data from seven Radiation Therapy Oncology Group (RTOG) trials indicated that higher radiotherapy dose intensities are associated with improved local-regional control and survival when chemoradiotherapy is administered [3]. Many phase I/II studies have demonstrated that dose escalation beyond the standard doses of 60–70 Gy is associated with better LC and improved overall survival in unresectable NSCLC cases, and the toxicities appear tolerable [4–6]. The recent success of stereotactic ablative radiotherapy for stage I NSCLC patients has renewed the interest in dose escalation [7]. However, the results of the RTOG 0617 clinical (Phase III) trial showed that there was no apparent survival benefit in the high-dose group [8]. The cardiopulmonary toxicities [9] and prolonged overall treatment time (OTT) were suspected as potential contributors [8].

Is there any other strategy for dose escalation in a post-RTOG 0617 era? The RTOG 0617 trial included three-dimensional conformal radiotherapy (3D-CRT) and standard intensity-modulated radiation therapy (IMRT), and higher doses (74 Gy) delivered to the planning tumor volume (PTV), including the subclinical area, may have been toxic to normal tissue. Moreover, dose escalation performed at 2 Gy per fraction leads to prolonged OTT and an increased risk of tumor cell repopulation during treatment. Thus, researchers have focused on intensity-modulated radiation therapy with a simultaneous integrated boost (SIB-IMRT), which is an advanced modality that delivers different dose levels in each treatment fraction, with a higher dose to the gross tumor and a relatively lower dose to the subclinical disease. The SIB technique can deliver a higher per-fraction dose and a higher total dose to the gross tumor volume (GTV) without prolonged OTT, and it can also maintain the per-fraction dose and total dose to the surrounding PTV at a level consistent with current practice [10]; thus, we hypothesize that this technique could represent an optional strategy for dose escalation in NSCLC. In our study, we determined the dosimetric feasibility of SIB-IMRT by comparing different SIB strategies with IMRT and three-dimensional conformal RT (3D-CRT).

## 2. Methods

### 2.1. Patient Selection

We reviewed 20 patients with biopsy-identified centrally located stage IIIA-IIIB NSCLC who were treated at the Hunan Cancer Hospital between 2013 and 2015. Patients with contralateral hilar or supraclavicular adenopathy were excluded. This retrospective study was approved by the Institutional Review Board, and the patients' identities were protected. The staging evaluations included bronchoscopy, positron emission tomography (PET)/CT, and brain magnetic resonance. The patient characteristics are described in Table 1.

### 2.2. Simulation

All of the patients underwent CT scanning (GE Lightspeed RT) in the supine position and were immobilized in an upper body cradle with their arms overhead. During the CT image acquisition, patient respiration was monitored with an external respiratory gating system (Active Breathing Coordinator™ R2.0; Elekta). The CT data were then imported into the planning system (Pinnacle, version 7.0, Philips).

### 2.3. Volume Definition

The gross tumor volume (GTV) was defined as any visible primary lesions and all lymph nodes > 1 cm in the short-axis dimension. The internal GTV (iGTV) was determined using a uniform 0.5 cm margin around the GTV for setup error. The spinal cord, heart, esophagus, and lungs were outlined as critical organs. The lung volume was outlined to exclude the iGTV. The clinical tumor volume (CTV) was determined by adding a 0.6 cm margin to the GTV, and the planning target volume (PTV) was determined by adding another 0.5 cm margin. A “simultaneous integrated avoidance (SIA)” volume was recommended to spare the esophagus, especially in the high-dose groups because the esophagus is always adjacent to the GTV. The SIA was adapted to maintain at least 5 mm of volume between the esophagus and the high-dose area when the esophagus and iGTV overlapped.

### 2.4. Dose Prescription

Five different dose plans (3D-CRT, IMRT, SIB-IMRT_2.3_, SIB-IMRT_2.5_, and SIB-IMRT_2.7_) were generated for each patient. All of the 3DCRT, IMRT, and SIB-IMRT plans were generated using the step-and-shoot technique with the Pinnacle planning system (Phillips Medical Systems). Beam arrangements were optimized for each of the 20 patients with the goal of reducing both the cardiac and pulmonary dose. All groups were prescribed a dose to the PTV of 60 Gy in 30 fractions. In the SIB groups, the prescribed doses to the iGTV were as follows: 69 Gy in 30 fractions at 2.3 Gy per fraction in the SIB-IMRT_2.3_ group; 75 Gy in 30 fractions at 2.5 Gy per fraction in the SIB-IMRT_2.5_ group; and 81 Gy in 30 fractions at 2.7 Gy per fraction in the SIB-IMRT_2.7_ group (Table 2).

The biological equivalent dose (BED) was calculated using the linear quadratic formula: BED = *nd*  × [1 + *d*/(*α*/*β*)], where *n* is the total number of fractions; *d* is the dose per fraction (Gy); and *α*/*β* = 10.

### 2.5. Plan Evaluation

The planning objectives were to give at least 95% of the prescribed doses to at least 95% of the PTVs while minimizing the irradiated volumes to the organs at risk. Dose volume histograms (DVHs) were calculated for each plan, and conformity was expressed by the conformity index (CI), which is defined as the volume encompassed by the 95% isodose divided by the PTV volume. The homogeneity index (HI) was defined as HI (D5%/D95%). Higher CI and lower HI values indicated a better conformity and homogeneity of the doses to the targets.

### 2.6. Statistical Analysis

Data were analyzed using the Statistical Package for Social Sciences (SPSS) Version 20.0. A paired samples *t*-test and two-way analysis of variance with a randomized block design were used to compare the dosimetric parameters, with statistical significance set at *P* < 0.05. The statistical tests were based on a two-sided significance level.

## 3. Results

### 3.1. Target Coverage

**Figure 1:**
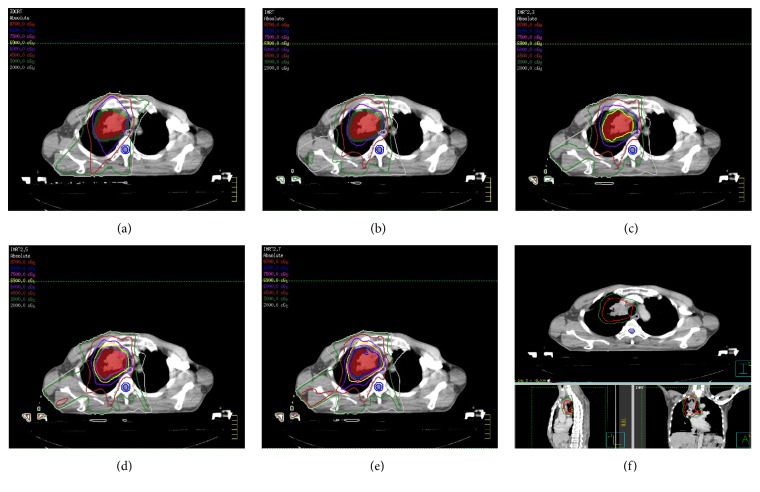
Dose distributions and target volumes of the five different treatment plans in the axial slices of one patient.* Notes*. (a–e) Dose distributions for the five different treatment plans for one patient. (a) 3DCRT plan, (b) IMRT plan, (c) SIB-IMRT_2.3_ plan, (d) SIB-IMRT_2.5_ plan, and (e) SIB-IMRT_2.7_ plan. (f) Target volume of the patient. 3DCRT, three-dimensional conformal radiotherapy; IMRT, intensity-modulated radiation therapy; SIB, simultaneous integrated boost.

The dose distributions for the five different treatment plans (3D-CRT, IMRT, SIB-IMRT_2.3_, SIB-IMRT_2.5_, and SIB-IMRT_2.7_) in the axial slices of one patient are shown in Figure 1. The isodoses were set from 20 to 87 Gy. The iGTV is outlined as a red area in all images, and the PTV is shown as the green area.

**Figure 2:**
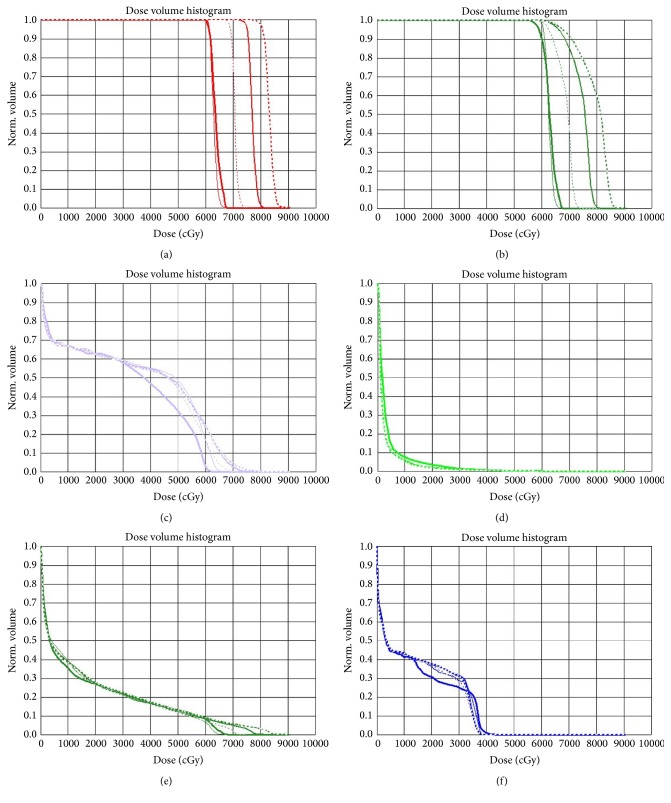
DVH of the target and the OARs of one patient with five different treatments.* Notes*. (a) DVH of the iGTV, (b) DVH of the PTV, (c) DVH of the esophagus, (d) DVH of the heart, (e) DVH of the lung, and (f) DVH of the spinal cord (line type: thick solid: 3DCRT; thin solid: IMRT; thin dashed: SIB-IMRT_2.3_; medium solid: SIB-IMRT_2.5_; and medium dashed: SIB-IMRT_2.7_). DVH, dose volume histogram; iGTV, internal gross tumor volume.

The DVH of one patient for the iGTV, the PTV, and the organs at risk (OARs; lung, heart, esophagus, and spinal cord) are demonstrated in Figure 2.

**Figure 3:**
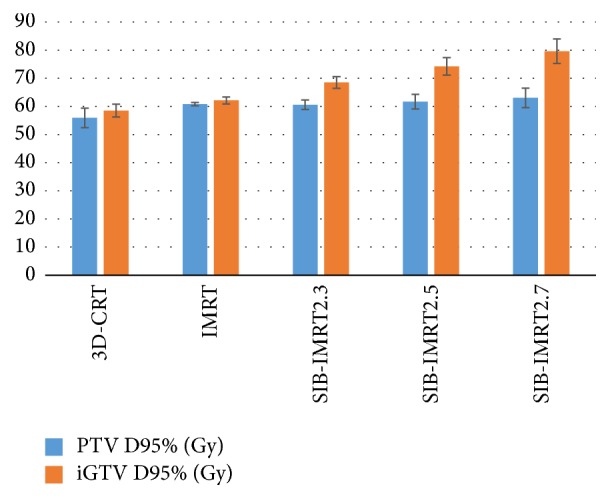
Total dose to the target volume in the 3D-CRT, IMRT, and SIB-IMRT groups.

In the SIB-IMRT groups, the iGTV D95% received 68.5 ± 2.1 Gy, 74.2 ± 3.1 Gy, and 79.6 ± 4.4 Gy (*P* < 0.05). The iGTV D95% was much higher in the SIB-IMRT groups than in the IMRT and 3D-CRT groups (*P* < 0.05). The PTV D95% in the 3D-CRT group received 55.9 ± 3.5 Gy, which was less than that of the other groups (*P* < 0.05). Significant differences in the PTV D95% were not observed among the IMRT groups (*P* > 0.05) (Table 3) (Figure 3).

The dose homogeneity was expressed by the HI, which is defined as HI D2%/D98%. The CI was defined as the volume encompassed by the 95% isodose divided by the PTV/iGTV volume.  Table 4 shows that the CI and HI for the PTV in the IMRT groups were better than those for the 3D-CRT group, although significant differences were not observed between the IMRT and SIB-IMRT groups. Significant differences were not observed in the CI and HI for the iGTV between the SIB-IMRT2.3 group and SIB-IMRT2.5 group, but these values are better than those in the SIB-IMRT2.7 group.

### 3.2. OAR

**Figure 4:**
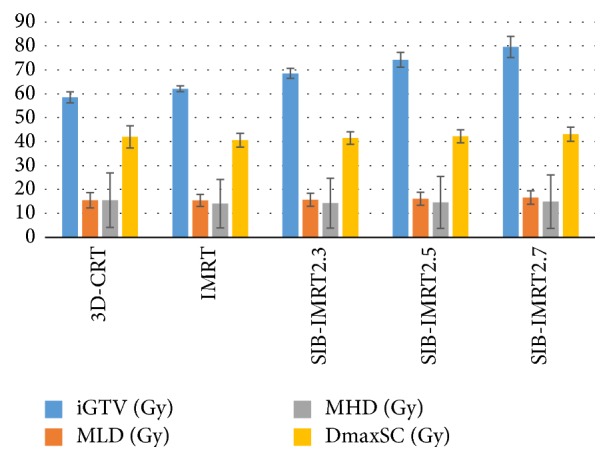
Dose comparison to the iGTV and OARs in the 3D-CRT, IMRT, and SIB-IMRT groups. iGTV, internal gross tumor volume; OAR, organ at risk; MLD, mean lung dose; MHD, mean heart dose; DmaxSC, maximum dose of the spinal cord.

The doses to the OARs in the different groups are shown in Table 5 and Figure 4. For the lungs, the SIB-IMRT2.7 group showed a significantly increased mean lung dose (MLD) (*P* < 0.05), although the limits were still met. A significant difference was observed in the lung V5 between the 3D-CRT and SIB-IMRT2.7 groups (*P* < 0.05), although slight differences were observed among the other groups (*P* > 0.05). The lung V20 was significantly higher in the 3D-CRT group than in the IMRT and SIB-IMRT groups. The lung maximum doses increased along with the prescribed dose to the iGTV in the SIB groups.

Significant differences were not observed in the Dmean and V50 to the esophagus, although the Dmax was increased in the SIB-IMRT groups. Significant differences were not observed in the Dmean and V40 to the heart.

The Dmax to the spinal cord was significant higher in the SIB-IMRT2.7 group than the other groups, although the limits were still met.

## 4. Discussion

In the present study, we compared five different dose plans (3D-CRT, IMRT, SIB-IMRT2.3, SIB-IMRT2.5, and SIB-IMRT2.7) to evaluate the dosimetric feasibility of dose escalation by SIB-IMRT in stage III NSCLC. Many studies have investigated dose escalation in NSCLC [4–6]; however, these studies did not focus on the SIB technique, which has been used to deliver higher doses to the GTV and standard doses to the PTV in the same fraction.

Although many studies have been published on the use of SIB-IMRT for head and neck carcinomas and anal carcinomas [11–13], the use of this technique for NSCLC is poorly understood. Swanick et al. [14] retrospectively considered the feasibility, toxicity, and failure patterns after hypofractionated SIB-IMRT (iGTV > 52.5 Gy and PTV > 45 Gy in 15 fractions) and found that it is a viable option for certain patients with NSCLC and presents limited overall high-grade toxicity. Han et al. [15] treated limited-disease small-cell lung cancer (LD-SCLC) with SIB-IMRT (prescribed dose to the GTV of 57 Gy at 1.9 Gy twice daily, dose to the CTV of 51 Gy at 1.7 Gy twice daily, and dose to the PTV of 45 Gy at 1.5 Gy twice daily) and concluded that SIB-IMRT was feasible and well tolerated in patients with LD-SCLC. Zhang et al. [16] analyzed a small cohort of special stage II (T2b-3N0M0) NSCLC patients with iGTV doses of 75 Gy, CTV doses of 60 Gy, and PTV doses 45 Gy in 15 fractions and found that SIB-IMRT is a potential option for special stage II (T2b-3N0M0) NSCLC that was medically inoperable.

As shown in Figure 1, we found that the doses delivered to the primary tumor (iGTV D95) were escalated by SIB-IMRT (68.5 Gy, 74.2 Gy, and 79.6 Gy, *P* < 0.05), whereas the PTV CI did not change. When the dose was escalated to 81 Gy in 2.7 Gy fractions, the iGTV CI and HI were worse than those of the SIB-IMRT2.5 and SIB-IMRT2.3 groups. However, doses escalated to more than 80 Gy could significantly influence the CI and HI.

An important reason why patients do not receive an adequate dose for tumor control is the limitation of normal tissue tolerance. Kong et al. [17] reported that lung toxicity was not associated with the dose prescribed to the tumor but was significantly (*P* < 0.001) correlated with normal lung dosimetric parameters, such as the MLD and V20. Bradley et al. [18] reported that an MLD-based model is better than other models for radiation pneumonitis. In our study, significant differences were not observed in the lung V5 and V20 among the IMRT groups. The MLD was significantly increased in the SIB-IMRT2.7 group, although it still met the limits. Although the V60 of the lung was also increased, reports on the relationship between the V60 and radiation-induced lung toxicity are not available. Thus, the efficacy and toxicity of the different groups should be assessed in clinical practice.

The heart V5, V30, V40, and spinal cord Dmax did not show significant differences under dose escalation. However, the esophagus Dmax was significantly increased with the prescribed dose, and the V60 of the heart was also increased.

In our retrospective study, although PET-CT and 4DCT examinations were not performed, the dosimetric feasibility of SIB-IMRT could still be demonstrated; however, PET-CT and 4DCT are highly recommended. Patients with contralateral hilar or supraclavicular adenopathy were excluded in this study; therefore, extensive stage III NSCLC requires further research. We believe that this dosimetric study lays a foundation for future clinical studies of dose escalation in locally advanced NSCLC, and our results indicate that advanced radiotherapy technologies are promising.

## 5. Conclusions

The results of our study demonstrate that SIB-IMRT allowed us to selectively increase the iGTV dose up to values of 69 Gy, 75 Gy, and 81 Gy without increased normal tissue exposure or prolonged OTT. Thus, escalating doses via SIB-IMRT is dosimetrically feasible, although it still warrants systematic evaluations by clinical trials. A randomized controlled phase II clinical trial to evaluate the clinical safety and efficacy of this approach is currently underway.

## Figures and Tables

**Table 1:** Patient characteristics (*N* = 20).

Characteristics	Value
Disease stage	
IIIA	10
IIIB	10
T stage	
T1	0
T2	8
T3	3
T4	9
N stage	
N0	0
N1	2
N2	13
N3	5
Histology	
Ade	3
Squa	17
Location	
Left	11
Right	9
iGTV (mm^3^)	
Mean	176.9
Range	88.0–296.7
PTV (mm^3^)	
Mean	468.6
Range	295.3–583.7

iGTV, internal gross tumor volume; PTV, planning tumor volume.

**Table 2:** Prescribed doses to the target volume.

	PTV			iGTV		
	Dose/F (Gy)	Total dose (Gy)	BED (Gy)	Dose/F (Gy)	Total dose (Gy)	BED (Gy)
3D-CRT	2.0	60	72	—	—	—
IMRT	2.0	60	72	—	—	—
SIB-IMRT_2.3_	2.0	60	72	2.3	69	84.87
SIB-IMRT_2.5_	2.0	60	72	2.5	75	93.75
SIB-IMRT_2.7_	2.0	60	72	2.7	81	102.9

3D-CRT, three-dimensional conformal radiotherapy; IMRT, intensity-modulated radiation therapy; SIB, simultaneous integrated boost.

**Table 3:** Dosimetric comparison of the 3D-CRT, IMRT, and SIB-IMRT groups to the target volumes.

	PTV D95% (Gy)	iGTV D95% (Gy)
3D-CRT	55.9 ± 3.5	58.5 ± 2.3
IMRT	60.8 ± 0.6	62.1 ± 1.2
SIB-IMRT_2.3_	60.6 ± 1.7	68.5 ± 2.1
SIB-IMRT_2.5_	61.7 ± 2.6	74.2 ± 3.1
SIB-IMRT_2.7_	63.0 ± 3.5	79.6 ± 4.4

3D-CRT, three-dimensional conformal radiotherapy; IMRT, intensity-modulated radiation therapy; SIB, simultaneous integrated boost.

**Table 4:** CI and HI comparison of the 3D-CRT, IMRT, and SIB-IMRT groups.

	PTV		iGTV	
	CI	HI	CI	HI
3D-CRT	0.945 ± 0.042^*∗*^	1.28 ± 0.17^*∗*^	—	—
IMRT	0.985 ± 0.017	1.14 ± 0.05	—	—
SIB-IMRT_2.3_	0.986 ± 0.015	—	0.998 ± 0.004	1.09 ± 0.03
SIB-IMRT_2.5_	0.985 ± 0.017	—	0.996 ± 0.006	1.10 ± 0.05
SIB-IMRT_2.7_	0.986 ± 0.017	—	0.990 ± 0.019^*∗∗*^	1.12 ± 0.07^*∗∗*^

^*∗*^ *P* < 0.05 compared with all other groups.

^*∗∗*^ *P* < 0.05 compared with the IMRT group.

**Table 5:** Dosimetric comparison of the 3D-CRT, IMRT, and SIB-IMRT groups to the organs at risk.

	3DCRT	IMRT	SIB-IMRT2.3	SIB-IMRT2.5	SIB-IMRT2.7
Lung					
Mean lung dose (Gy)	15.5 ± 3.2	15.4 ± 2.5	15.7 ± 2.7	16.1 ± 2.7	16.6 ± 2.8^*∗*^
V5 (%)	46.4 ± 12.9	49.2 ± 9.7	49.1 ± 10.1	49.3 ± 10.6	49.9 ± 10.7^*∗∗*^
V20 (%)	29.5 ± 7.3^*∗*^	26.9 ± 4.3	27.1 ± 4.5	27.3 ± 4.7	28.0 ± 5.1
V60 (%)	2.9 ± 2.1^*∗*^	1.3 ± 1.6	5.6 ± 2.1	6.7 ± 2.4^*∗*^	7.7 ± 2.6^*∗*^
Heart					
Mean dose (Gy)	15.5 ± 11.4	14.1 ± 10.1	14.3 ± 10.4	14.6 ± 10.9	14.9 ± 11.2
V5 (%)	47.2 ± 32.1	59.6 ± 33.4	50.6 ± 33.8	49.3 ± 34.2	50.0 ± 33.9
V40 (%)	14.5 ± 14.7	11.9 ± 11.0	11.9 ± 11.1	12.3 ± 11.5	12.7 ± 11.9
V60 (%)	4.7 ± 6.3	2.12 ± 2.6^*∗*^	3.1 ± 3.5	4.1 ± 4.9	4.6 ± 5.6
Eso					
Mean dose (Gy)	25.7 ± 9.9	26.6 ± 9.0	26.8 ± 9.4	26.9 ± 9.6	27.4 ± 9.8
V50 (%)	26.6 ± 18.3	27.0 ± 17.1	26.5 ± 16.4	25.9 ± 16.3	26.1 ± 16.2
Maximum	62.4 ± 3.7	64.0 ± 2.3	68.0 ± 3.8^*∗*^	73.0 ± 5.5^*∗*^	77.6 ± 6.9^*∗*^
Spinal cord					
Maximum	42.0 ± 4.6	40.6 ± 2.9	41.5 ± 2.6	42.2 ± 2.7	43.1 ± 2.9^*∗∗∗*^

^*∗*^ *P* < 0.05 compared with all other groups.

^*∗∗*^ *P* < 0.05 compared with the 3DCRT group.

^*∗∗∗*^ *P* < 0.05 compared with the other IMRT groups.
